# Subchronic tolerance trials of graded oral supplementation with ornithine hydrochloride or citrulline in healthy adults

**DOI:** 10.1007/s00726-022-03227-4

**Published:** 2022-12-26

**Authors:** Naoki Miura, Koji Morishita, Takamasa Yasuda, Saori Akiduki, Hideki Matsumoto

**Affiliations:** 1 Miura Medical Clinic, Higashitenma, Osaka, Japan; 2 Japan Branch of International Council for Amino Acid Science (ICAAS), Hatchobori, Tokyo, Japan

**Keywords:** NOAEL, Citrulline, Dietary supplements, Human, Ornithine, Safety

## Abstract

Ornithine and citrulline are amino acids used in dietary supplements and nutritional products consumed by healthy consumers, but the safe supplementation levels of these compounds are unknown. The objective of this study was to conduct two 4-week clinical trials to evaluate the safety and tolerability of graded dosages of oral ornithine (as hydrochloride) and citrulline. Healthy male adults (*n* = 60, age 41.4 ± 1.5 years) completed graded dosages of either ornithine hydrochloride (3.2, 6, 9.2, and 12 g/day) or citrulline (6, 12, 18, and 24 g/day) supplement for 4 weeks with 2-week wash-out periods in between. Primary outcomes included vitals, a broad spectrum of circulating biochemical analytes, body weight, sleep quality, and mental self-assessment. In the ornithine hydrochloride supplementation group, minor increase in plasma aspartic acid and glutamic acid concentrations was observed at the highest intake dosages. In the citrulline supplementation group, minor changes in laboratory data for serum lactate dehydrogenase and plasma amino acid concentration of lysine, methionine, threonine, aspartic acid, glutamic acid, glutamine and ornithine, arginine, and citrulline itself were measured. No other changes in measured parameters were observed, and study subjects tolerated 4-week-long oral supplementation of ornithine hydrochloride or citrulline without treatment-related adverse events. A clinical, no-observed-adverse-effect-level (NOAEL) of ornithine hydrochloride and citrulline supplementation in healthy adult males was determined to be 12 g/day and 24 g/day (4 weeks), respectively.

## Introduction

L-ornithine (ORN) and L-citrulline (CIT) are amino acids present in nutritional sources as free amino acids but are not found in proteins (Wu 2021). ORN is naturally present in several food sources, such as brackish-water Bivalve (175.8 mg/100 g), scallop (7.0 mg/100 g), little-neck clam (6.7 mg/100 g) (Uchisawa et al. 2004), mahi-mahi (2.7–5.5 mg/100 g), yellowfin tuna (0.8–7.2 mg/100 g), American red snapper (0.5–1.7 mg/100 g) (Antoine et al. 2001), and green tea (5.1 mg/100 g) (Ohtsuki et al. 1987). CIT is present in watermelon (180 mg/100 g), loofah (57 mg/100 g), melon (50 mg/100 g), bitter gourd (16 mg/100 g), and cucumber (9.6 mg/100 g) (Hayashi 2008). In addition, both ORN not CIT are frequently added to dietary supplements for various purposes (Moinard et al. 2008; Sugino et al. 2008; Dietary Supplement Label Database 2022). For example, ORN is used in dietary supplements as an agent to improve liver function (Müting et al. 1992), as a sleep enhancer (Horiuchi et al. 2013; Miyake et al. 2014), or as a precursor to polyamine production and subsequent collagen formation for bone healing (Meesters et al. 2020; Wijnands et al. 2012). CIT has been claimed to aid in recovery from fatigue or exercise (Bendahan et al. 2002; Perez-Guisado et al. 2010) and to reduce swelling (Morita et al. 2012). In recent years, some human studies of CIT supplementation have been reported about protein metabolism under malnutrition (Jourdan et al 2015; Bouillane et al. 2019) and high-intensity interval training (Marcangeli et al 2022). Despite their intake from dietary supplements, there have been few studies on safe chronic or subchronic dose. A subacute study on the safety on ORN hydrochloride supplementation, in which 16 healthy adults ingested 3 g/day, 3 month trial, showed nocausal adverse effects at tested dose (Morita et al. 2013). An acute study on the safety on CIT supplementation, in which 8 healthy adults ingested 2, 5, 10, and 15 g/day, showed no causal adverse effects at any tested dose (Moinard et al. 2008) and 17 healthy adult males ingesting 6 g/day of CIT in repeated 4-week trials also demonstrated no adverse effects (Figueroa et al. 2010).

Given that ORN and CIT are used as supplementation, and the lack of adequate clinical safety data, the primary objective of this study was to determine the clinical no-observed adverse effect level (NOAEL) for a 4-week intake of graded doses of ORN at 3.2–12 g/day and CIT at 6–24 g/day, based on the previously established methodology applied to other amino acids (Cynober et al. 2012; Cynober et al. 2016; Deutz et al. 2017; Cynober et al. 2020; Gheller et al. 2020; Blachier F et al. 2021). The limited data available on biomarkers for clinical toxicity of either ORN or CIT led us to use changes in a broad range of circulating biochemical analyte concentrations, anthropometry, macronutrient and caloric intakes, sleep quality, mental fatigue, and any treatment-specific adverse effects as indicators of tolerance to increasing doses of both amino acids. The initial dose of ORN and CIT in each dose series was the supplemental doses deemed as safe for subchronic intake (3.2 g/day and 6 g/day, respectively).

## Materials and methods

### Ethical review

The procedures in the two clinical trials were in accordance with ethical standards and were approved by the Miura Medical Clinic Ethics Review Committee (ethical approval code R1913 of 21 February 2020). The trials were registered in the clinical trial registry system operated by the University Hospital Medical Information Network (UMIN-CTR) under UMIN 000,043,616 (15 March 2021). In conducting this trial, the 1.5th edition of the protocol was prepared on 9 June 2021, to include COVID-19 infection control. The study was approved by the ethics committee on 21 June 2021.

### Subjects

The study population consisted of 60 healthy male subjects (*n* = 60). This study was conducted at Miura Medical Clinic (Osaka, Japan) between April 2021 and November 2021. Subject recruitment, blood collection, and anthropometric and body composition measurements were conducted in the Miura Medical Clinic and managed by a project coordinator with the proper training (Oneness Support Ltd., Osaka, Japan). The subjects provided written, informed consent before participation. Participants were publicly recruited in the Osaka city area (Japan) and divided into two separate groups of 30 subjects, one receiving graded doses of ornithine and the other receiving graded doses of citrulline.

### Screening criteria

During screening, all participants completed a health history and physical activity questionnaire that included current and recent medications and supplement use. Inclusion criteria were as follows: healthy males aged 20 to less than 60 years at the time of informed consent, those whose written informed consent was obtained after a thorough understanding of the purpose and content of this study, and able to swallow multiple capsules. Exclusion criteria were as follows: (1) continuously consuming or possibly consuming medicinal products during the trial supplement intake period, (2) consuming supplements containing amino acid components from the time of participation in the study until the end of the trial supplement intake period, (3) involved in other human clinical studies or who would be participating in this study within 4 weeks after the end of the other studies, or who were scheduled to participate in other studies during this study period, (4) cardiac, liver, and kidney disease, respiratory disease, history of cardiovascular disease, diabetes mellitus, any intestinal disease/enteropathies, undergoing treatment for or have a history of serious illnesses such as cancer or tuberculosis, (5) allergies to food or drugs, and (6) others who are determined by the appointed medical staff to be unsuitable to include in the study.

### Supplementation

The two trials consisted of four graded doses of cellulose encapsulated ORN hydrochloride or CIT (one capsule, 0.4 g). Both amino acids were produced by Kyowa Hakko Bio Co., Ltd. (Tokyo, Japan). The starting dose (ORN 3.2 g/day, CIT 6 g/day) was chosen according to previous reported intake doses of human ingestion studies. This initial supplemental dose was assumed to be safe, since previous studies set the experimental intakes of ORN or CIT at more than 8 g/day or 6 g/day without noting significant adverse effects, respectively (Moinard et al. 2008; Figueroa et al. 2010; Morita et al. 2013). Subsequent doses were determined based on experience with other amino acids (Deutz et al. 2017; Gheller et al. 2020; Miura et al. 2021) as approximated multiple of the starting dose (ORN 3.2, 6, 9.2, and 12 g/day, CIT 6, 12, 18, and 24 g/day). Participants continued to the next higher doses following a 2-week wash-out period at the discretion of the investigator.

### Study intervention, sample collection, and analysis

Subjects received supplements for the whole 4-week test period and were instructed to consume them daily before 12:00 pm, consistent with previous research (Gheller et al. 2020; Miura et al. 2021). At the end of each 4-week test period, the subjects visited the clinic for safety evaluation following overnight fasting for at least 8 h. Laboratory measurements included body weight, blood pressure, and pulse rate. All biochemical analyses were conducted by LSI Medience Co., Ltd. (Osaka, Japan) using routine clinical methodologies. Biochemical serum analyses included white and red blood cell counts measured by an automated flow-cytometry analyzer (XE-2100, Sysmex Co., Tokyo, Japan), hemoglobin, and hematocrit and platelet count measured by the red blood cell pulse peak detection method and the electrical resistance detection method, respectively (XE-2100, Sysmex Co.). Blood chemistry (glutamic oxaloacetic transaminase, glutamic pyruvic transaminase, gamma-glutamyltransferase, alkaline phosphatase, lactate dehydrogenase, total bilirubin, creatinine, blood urea nitrogen, triglycerides, glucose, uric acid, creatine phosphokinase, and total protein) was evaluated based on the methodologies recommended by the Japanese Society of Clinical Chemistry using a Hitachi automated analyzer (Labospect LST008α, Hitachi Co., Tokyo, Japan) combined with a JCA-BM8000 automatic analyzer (JEOL Co., Inc., Tokyo, Japan). Albumin was analyzed by the dye binding-bromocresol purple method using the Labospect LST008α, and the serum levels of electrolytes (sodium, potassium, chloride, and calcium) were analyzed by the electrode method using the same instrument. Blood glucose was measured enzymatically using the JCA-BM9130 automatic analyzer (JEOL Co., Inc.). Plasma concentrations of free amino acids were measured by pre-column derivatization high-performance liquid chromatography (Agilent Co., Santa Clara, CA, USA).

Compliance (intake of the tested amino acids) was assessed daily through an online survey. Another survey was used to measure relative changes in subjective sleep quality and mental fatigue using a linear scale ranging from − 5 to + 5, with zero corresponding to “no change” from the previous day, negative values indicating worsening, and positive values indicating improving. In addition, a dietary survey using the Sasaki Food Habit Assessment (Sasaki et al. 2003; Kobayashi et al. 2011) was conducted at the end of each 4-week test period to assess food intake status in terms of total food energy and main macronutrient categories (proteins, lipids, and carbohydrates). An individual adverse event was defined as an independent event when its endpoint was confirmed by the investigator. Gastrointestinal symptoms, side effects of COVID-19 vaccination, “cold-like” symptoms, headache, fatigue, exanthema, joint pain, hematuria, drowsy, giddiness, and stomatitis were considered adverse events. Those events were assessed as unrelated, probably related, or related to the test compound by the investigator at the end of each 4-week test period.

### Statistical analyses

The statistical analyses were performed on the per-protocol set (PPS). All results are presented as means and standard errors (SEM). Significant differences were determined using one-factor repeated-measures ANOVA followed by Tukey’s multiple group comparison significance test (SPSS Ver. 26, Japan IBM Co. Ltd., Tokyo, Japan). The level of statistical significance was determined at *p* < 0.05.

## Results

### Subjects

**Fig. 1:**
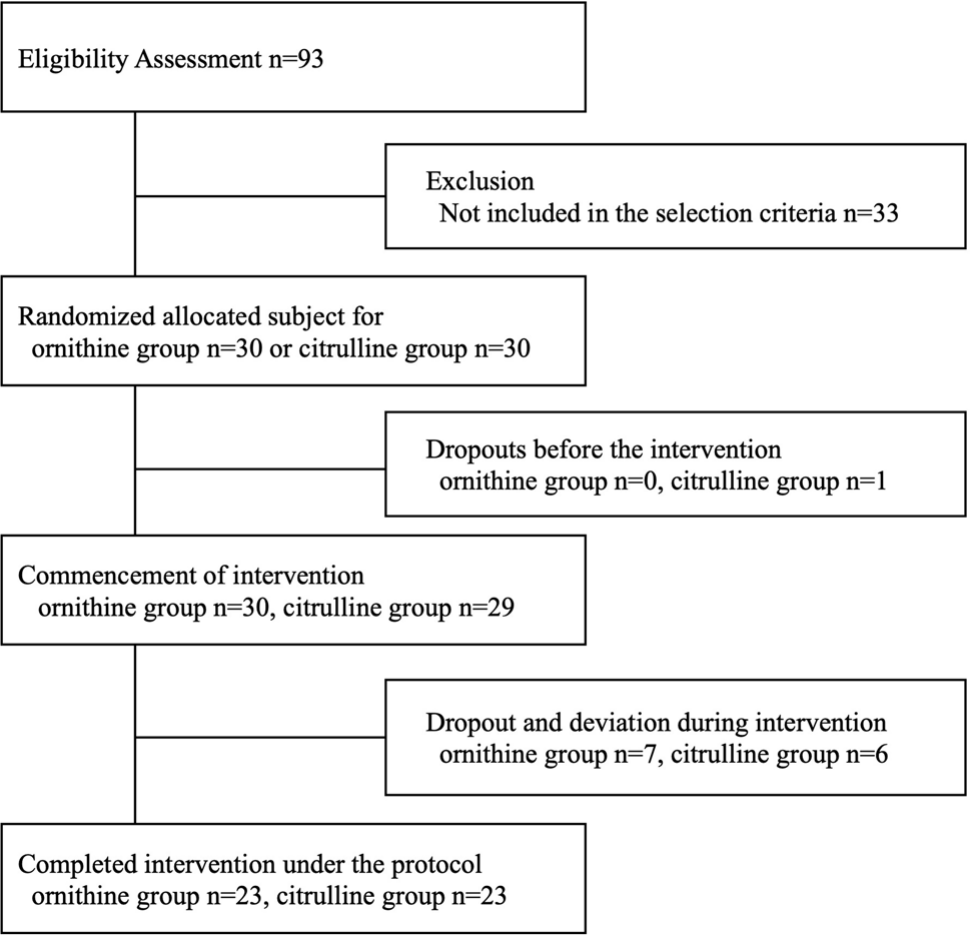
Flow diagram of participants through each step in this study

The study subjects were 60 healthy adult males who met the screening criteria. Data of the screened subject characteristics are shown in Table 1. The subjects were randomly divided into ORN or CIT intake groups based on their body weight (Table 1). There was no statistically significant difference between divided data. In the ORN group, 4 of the initial 30 subjects were withdrawn at the discretion of the investigator after receiving requests by the subjects due to causes unrelated to the study (one subject (ID13351) at the end of the 3.2 g/day × 28 days term and three subjects (ID13327, ID13329, and ID13362) at the end of the 6 g/day × 28 days term). In addition, the investigator judged those 3 subjects (ID13383 at the end of the 3.2 g/day × 28 days term, ID13328 at the end of the 6 g/day × 28 days term, and ID13384 at the end of the 12 g/day × 28 days term) had breached the consumption protocol for ORN capsule intake. Therefore, 23 subjects in the ORN group were evaluated in the per-protocol set (Fig. 1). In the CIT group, 3 of the initial 30 subjects were withdrawn at the discretion of the investigator after receiving requests by the subjects due to causes unrelated to the study (one subject (ID13348) before the start of the intake, one subject (ID13378) at the end of the 6 g/day × 28 days term and one subject (ID13344) at the end of the 12 g/day × 28 days term). In addition, the investigator judged those 4 subjects (ID13388 at the end of the 6 g/day × 28 days term, ID13349 at the end of the 6 g/day × 28 days term, and ID13331, and ID13391 at the end of the 24 g/day × 28 days term) had breached the consumption protocol for CIT capsule intake. Therefore, 23 subjects in the CIT group were evaluated in the per-protocol set (Fig. 1).

**Table 1:** Characteristics data of the study participants

		Whole subjects		Ornithine group		Citrulline group	
		*n*	mean ± SE	*n*	mean ± SE	*n*	mean ± SE
Age	(year)	60	41.4 ± 1.5	30	41.4 ± 2.1	30	41.5 ± 2.1
BH	(cm)	60	172.1 ± 0.7	30	171.2 ± 1.0	30	173.0 ± 1.1
BW	(Kg)	60	70.2 ± 1.0	30	69.8 ± 1.5	30	70.6 ± 1.5
BMI	(kg/m^2^)	60	23.7 ± 0.3	30	23.8 ± 0.4	30	23.6 ± 0.5
SAP	(mmHg)	60	122.4 ± 1.3	30	121.3 ± 1.8	30	123.5 ± 1.9
DBP	(mmHg)	60	73.8 ± 1.1	30	72.7 ± 1.7	30	75.0 ± 1.3
HR	(bpm)	60	67.5 ± 1.2	30	67.9 ± 1.8	30	67.1 ± 1.5

Values represent the mean and standers error (SE)

*BH* body height, *BW* body weight, *BMI* body mass index, *SAP* systolic arterial pressure, *DBP* diastolic blood pressure, HR heart rate

### Clinical laboratory test

The results of biochemical analyses conducted at the end of each 4-week test period are shown in Table 2. In the ORN administration group, blood parameters were not statistically significantly different when compared with baseline values. CIT administration triggered a small but statistically significant increase in lactate dehydrogenase (CIT doses of 12 and 18 g/day) when compared to the baseline values, but these changes were within the reference ranges of the clinic and were not to be toxicologically relevant. No other changes in blood parameters were observed. In addition, in one subject (ID13387), a single point value of creatine kinase (CIT doses 6 g/day) was above the reference range. The changed value was not dose-dependent, and a medical interview and recovery within the reference range after measurements resulted in this, observations not being considered a causal pathological change by the lead investigator.

**Table 2:** Changes in biochemical blood parameters throughout the oral ornithine or citrulline supplementation period in healthy adults

Blood parameter	Ornithine (*n* = 23)		Citrulline (*n* = 23)	
Total protein	Baseline	7.1 ± 0.1	Baseline	7.2 ± 0.1
Ref. 6.7–8.3	3.2 g/day	7.0 ± 0.1	6 g/day	7.2 ± 0.1
(g/dL)	6 g/day	7.2 ± 0.1	12 g/day	7.2 ± 0.1
	9.2 g/day	7.2 ± 0.1	18 g/day	7.1 ± 0.1
	12 g/day	7.3 ± 0.1	24 g/day	7.3 ± 0.1
Albumin	Baseline	4.6 ± 0.0	Baseline	4.6 ± 0.1
Ref. 3.8–5.2	3.2 g/day	4.5 ± 0.1	6 g/day	4.5 ± 0.1
(g/dL)	6 g/day	4.5 ± 0.0	12 g/day	4.5 ± 0.1
	9.2 g/day	4.6 ± 0.1	18 g/day	4.5 ± 0.1
	12 g/day	4.6 ± 0.1	24 g/day	4.6 ± 0.1
Total bilirubin	Baseline	0.97 ± 0.06	Baseline	0.89 ± 0.06
Ref. 0.2–1.2	3.2 g/day	1.01 ± 0.09	6 g/day	0.88 ± 0.06
(mg/dL)	6 g/day	0.96 ± 0.08	12 g/day	0.90 ± 0.07
	9.2 g/day	0.94 ± 0.08	18 g/day	0.83 ± 0.06
	12 g/day	1.01 ± 0.07	24 g/day	0.84 ± 0.05
AST (GOT)	Baseline	21.5 ± 1.0	Baseline	20.4 ± 0.9
Ref. 10–40	3.2 g/day	22.2 ± 1.6	6 g/day	20.7 ± 1.2
(U/L)	6 g/day	21.0 ± 1.1	12 g/day	25.9 ± 5.4
	9.2 g/day	20.6 ± 1.1	18 g/day	22.1 ± 1.6
	12 g/day	21.9 ± 1.3	24 g/day	20.9 ± 0.9
ALT (GPT)	Baseline	21.9 ± 2.3	Baseline	18.8 ± 1.4
Ref. 5–45	3.2 g/day	21.5 ± 2.1	6 g/day	19.0 ± 2.0
(U/L)	6 g/day	21.2 ± 2.6	12 g/day	21.0 ± 2.7
	9.2 g/day	21.7 ± 2.7	18 g/day	19.6 ± 1.9
	12 g/day	23.4 ± 3.1	24 g/day	18.3 ± 1.2
ALP	Baseline	191.6 ± 12.0	Baseline	196.0 ± 9.3
Ref. 100–325	3.2 g/day	179.7 ± 10.0	6 g/day	185.6 ± 10.3
(U/L)	6 g/day	181.9 ± 8.4	12 g/day	186.2 ± 12.6
	9.2 g/day	184.9 ± 8.0	18 g/day	198.1 ± 15.2
	12 g/day	197.4 ± 9.2	24 g/day	193.5 ± 12.5
Gamma-GT	Baseline	24.8 ± 2.2	Baseline	25.6 ± 2.7
Ref. less than 80	3.2 g/day	24.9 ± 2.5	6 g/day	26.6 ± 3.2
(U/L)	6 g/day	25.4 ± 2.4	12 g/day	24.3 ± 2.5
	9.2 g/day	24.4 ± 2.2	18 g/day	24.3 ± 2.4
	12 g/day	25.8 ± 2.4	24 g/day	24.8 ± 2.6
LDH	Baseline	181.7 ± 5.2	Baseline	171.0 ± 4.4
Ref. 120–240	3.2 g/day	191.4 ± 6.3	6 g/day	182.4 ± 4.8
(U/L)	6 g/day	195.2 ± 5.5	12 g/day	193.5 ± 4.8*
	9.2 g/day	189.4 ± 5.2	18 g/day	191.5 ± 5.9*
	12 g/day	193.4 ± 5.4	24 g/day	190.1 ± 5.5
CK	Baseline	138.7 ± 12.1	Baseline	164.5 ± 16.4
Ref. 60–270	3.2 g/day	248.3 ± 95.3	6 g/day	198.4 ± 33.7
(U/L)	6 g/day	155.7 ± 14.7	12 g/day	434.0 ± 250.1
	9.2 g/day	140.9 ± 11.3	18 g/day	251.8 ± 53.9
	12 g/day	137.0 ± 8.7	24 g/day	177.7 ± 18.1
Blood urea nitrogen	Baseline	15.1 ± 0.9	Baseline	14.1 ± 0.6
Ref. 8.0–20	3.2 g/day	14.0 ± 0.8	6 g/day	13.5 ± 0.5
(mg/dL)	6 g/day	15.0 ± 0.8	12 g/day	15.6 ± 0.6
	9.2 g/day	14.5 ± 0.6	18 g/day	15.1 ± 0.5
	12 g/day	14.1 ± 0.7	24 g/day	15.8 ± 0.4
Creatine	Baseline	0.85 ± 0.02	Baseline	0.84 ± 0.02
Ref. 0.61–1.04	3.2 g/day	0.85 ± 0.02	6 g/day	0.87 ± 0.02
(mg/dL)	6 g/day	0.81 ± 0.02	12 g/day	0.86 ± 0.02
	9.2 g/day	0.84 ± 0.02	18 g/day	0.86 ± 0.02
	12 g/day	0.81 ± 0.02	24 g/day	0.87 ± 0.02
Uric acid	Baseline	5.9 ± 0.2	Baseline	5.7 ± 0.2
Ref. 3.8–7.0	3.2 g/day	5.6 ± 0.2	6 g/day	5.8 ± 0.2
(mg/dL)	6 g/day	5.6 ± 0.2	12 g/day	5.7 ± 0.2
	9.2 g/day	5.6 ± 0.2	18 g/day	5.5 ± 0.2
	12 g/day	5.7 ± 0.2	24 g/day	5.5 ± 0.2
Sodium	Baseline	141.0 ± 0.3	Baseline	140.9 ± 0.2
Ref. 137–147	3.2 g/day	141.2 ± 0.4	6 g/day	140.7 ± 0.2
(mmol/L)	6 g/day	141.1 ± 0.3	12 g/day	141.0 ± 0.2
	9.2 g/day	140.5 ± 0.2	18 g/day	140.5 ± 0.3
	12 g/day	141.0 ± 0.2	24 g/day	140.7 ± 0.3
Potassium	Baseline	4.2 ± 0.0	Baseline	4.3 ± 0.0
Ref. 3.5–5.0	3.2 g/day	4.2 ± 0.1	6 g/day	4.2 ± 0.1
(mmol/L)	6 g/day	4.1 ± 0.0	12 g/day	4.1 ± 0.0
	9.2 g/day	4.2 ± 0.1	18 g/day	4.2 ± 0.0
	12 g/day	4.2 ± 0.1	24 g/day	4.3 ± 0.0
Chloride	Baseline	102.4 ± 0.4	Baseline	102.7 ± 0.4
Ref. 98–108	3.2 g/day	103.0 ± 0.5	6 g/day	102.9 ± 0.3
(mmol/L)	6 g/day	104.0 ± 0.4	12 g/day	103.7 ± 0.4
	9.2 g/day	103.9 ± 0.4	18 g/day	103.8 ± 0.4
	12 g/day	103.1 ± 0.5	24 g/day	102.4 ± 0.3
Calcium	Baseline	9.4 ± 0.0	Baseline	9.4 ± 0.1
Ref. 8.4–10.4	3.2 g/day	9.3 ± 0.1	6 g/day	9.3 ± 0.1
(mg/dL)	6 g/day	9.2 ± 0.1	12 g/day	9.3 ± 0.1
	9.2 g/day	9.3 ± 0.0	18 g/day	9.3 ± 0.1
	12 g/day	9.5 ± 0.1	24 g/day	9.5 ± 0.0
Total cholesterol	Baseline	207.3 ± 6.7	Baseline	208.3 ± 6.7
Ref. 210–219	3.2 g/day	201.7 ± 6.3	6 g/day	194.3 ± 5.9
(mg/dL)	6 g/day	198.0 ± 6.7	12 g/day	192.0 ± 6.0
	9.2 g/day	198.4 ± 7.8	18 g/day	193.0 ± 6.7
	12 g/day	205.8 ± 6.7	24 g/day	201.7 ± 7.3
Triglycerides	Baseline	96.1 ± 7.0	Baseline	84.5 ± 8.9
Ref. 30–149	3.2 g/day	97.4 ± 11.2	6 g/day	85.1 ± 9.2
(mg/dL)	6 g/day	95.8 ± 11.0	12 g/day	85.2 ± 11.6
	9.2 g/day	90.8 ± 9.0	18 g/day	76.0 ± 10.7
	12 g/day	94.3 ± 8.2	24 g/day	72.4 ± 7.8
HDL cholesterol	Baseline	62.9 ± 2.6	Baseline	68.8 ± 3.1
Ref. 40–85	3.2 g/day	58.7 ± 2.6	6 g/day	64.6 ± 2.8
(mg/dL)	6 g/day	59.0 ± 2.3	12 g/day	63.3 ± 2.7
	9.2 g/day	59.1 ± 2.4	18 g/day	64.7 ± 2.7
	12 g/day	60.4 ± 2.4	24 g/day	67.4 ± 2.7
LDL cholesterol	Baseline	124.8 ± 6.4	Baseline	122.4 ± 5.3
Ref. 65–139	3.2 g/day	123.6 ± 6.1	6 g/day	113.1 ± 4.5
(mg/dL)	6 g/day	118.9 ± 6.3	12 g/day	111.4 ± 4.5
	9.2 g/day	119.3 ± 7.4	18 g/day	111.2 ± 5.1
	12 g/day	121.3 ± 6.6	24 g/day	113.8 ± 5.4
Glucose	Baseline	87.0 ± 1.1	Baseline	85.6 ± 1.3
Ref. 70–109	3.2 g/day	87.8 ± 1.5	6 g/day	88.2 ± 1.2
(mg/dL)	6 g/day	86.9 ± 1.8	12 g/day	87.7 ± 2.4
	9.2 g/day	88.4 ± 1.5	18 g/day	89.8 ± 2.3
	12 g/day	88.2 ± 1.3	24 g/day	88.6 ± 1.2
White blood cells	Baseline	5800 ± 341	Baseline	5083 ± 160
Ref. 3300–9000	3.2 g/day	5430 ± 313	6 g/day	5609 ± 294
(/µL)	6 g/day	5526 ± 265	12 g/day	5370 ± 297
	9.2 g/day	5665 ± 305	18 g/day	5613 ± 330
	12 g/day	5409 ± 228	24 g/day	5417 ± 244
Red blood cells	Baseline	503.0 ± 5.5	Baseline	497.8 ± 6.2
Ref. 430–580	3.2 g/day	497.7 ± 6.5	6 g/day	491.5 ± 6.7
(× 10^4^/µL)	6 g/day	489.4 ± 7.2	12 g/day	482.9 ± 6.7
	9.2 g/day	492.2 ± 6.6	18 g/day	484.4 ± 6.6
	12 g/day	500.1 ± 6.8	24 g/day	498.6 ± 7.5
Hemoglobin	Baseline	15.3 ± 0.2	Baseline	15.0 ± 0.2
Ref. 13.5–17.5	3.2 g/day	15.0 ± 0.2	6 g/day	14.7 ± 0.2
(g/dL)	6 g/day	14.9 ± 0.2	12 g/day	14.7 ± 0.2
	9.2 g/day	14.9 ± 0.2	18 g/day	14.7 ± 0.2
	12 g/day	15.2 ± 0.2	24 g/day	15.1 ± 0.2
Hematocrit	Baseline	47.3 ± 0.4	Baseline	46.4 ± 0.5
Ref. 39.7–52.4	3.2 g/day	47.2 ± 0.5	6 g/day	46.7 ± 0.6
(%)	6 g/day	46.8 ± 0.5	12 g/day	45.9 ± 0.6
	9.2 g/day	46.9 ± 0.5	18 g/day	45.9 ± 0.5
	12 g/day	47.1 ± 0.5	24 g/day	46.8 ± 0.5
Platelet	Baseline	26.2 ± 0.9	Baseline	27.4 ± 1.0
Ref. 14–34	3.2 g/day	26.0 ± 0.8	6 g/day	27.5 ± 1.1
(× 10^4^/µL)	6 g/day	26.6 ± 0.9	12 g/day	26.7 ± 0.9
	9.2 g/day	26.3 ± 0.9	18 g/day	26.8 ± 1.1
	12 g/day	26.6 ± 0.9	24 g/day	27.0 ± 1.1

Values represent the mean and standers error (SE) (*n* = 23). One-way ANOVA was followed with Tukey’s multiple comparisons test between each amino acid intake: *p < 0.05 from baseline

*AST* aspartate transaminase, *GOT* glutamic oxaloacetic transaminase, *ALT* alanine aminotransferase, *GPT* glutamic pyruvic transaminase, *ALP* Alkaline phosphatase, *gamma-GT* gamma-glutamyltransferase, *LDH* lactate dehydrogenase, *CK* creatine kinase, *Ref.* reference range

The results of amino acids analyses conducted at the end of each 4-week test period are shown in Table 3. Plasma concentrations of the essential amino acids, non-essential amino acids, ORN, and CIT were measured. In the ORN supplementation group, statistically significant increases in plasma concentration of aspartic acid (ORN doses, 6.2 and 9 g/day), glutamic acid (ORN doses, 6 g/day), and ORN (ORN doses, 12 g/day) were observed. In the CIT supplementation group, statistically significant decreases in plasma concentrations of lysine (CIT doses, 6, 12, 18, and 24 g/day), methionine (CIT doses, 24 g/day), threonine (CIT doses, 18 and 24 g/day), and glutamine (CIT doses, 6, 12 and 18 g/day) were found. In addition, in the CIT group, a statistically significant increase in arginine (CIT doses, 12, 18 and 24 g/day), aspartic acid (CIT doses, 12 g/day), glutamic acid (CIT doses, 12 and 18 g/day), ORN (CIT doses, 12, 18 and 24 g/day), and CIT (CIT doses, 18 and 24 g/day) was detected. No other changes in plasma amino acid concentrations were observed.

**Table 3:** Changes in plasma free amino acid levels throughout the oral ornithine or citrulline supplementation period in healthy adults

EAA (μmol/L)	Ornithine (*n* = 23)		Citrulline (*n* = 23)	
Histidine	Baseline	79.9 ± 1.5	Baseline	80.2 ± 1.6
Ref. 67.9–97.1	3.2 g/day	79.6 ± 1.2	6 g/day	77.2 ± 1.9
	6 g/day	79.2 ± 1.8	12 g/day	78.5 ± 1.7
	9.2 g/day	80.0 ± 1.5	18 g/day	76.5 ± 1.4
	12 g/day	79.9 ± 1.8	24 g/day	77.1 ± 1.7
Isoleucine	Baseline	67.1 ± 3.3	Baseline	60.3 ± 2.5
Ref. 41.3–84.9	3.2 g/day	65.9 ± 2.5	6 g/day	56.0 ± 1.7
	6 g/day	67.2 ± 2.6	12 g/day	60.0 ± 3.5
	9.2 g/day	67.8 ± 2.2	18 g/day	59.4 ± 2.4
	12 g/day	66.6 ± 2.8	24 g/day	53.8 ± 2.3
Leucine	Baseline	127.6 ± 5.6	Baseline	115.3 ± 3.7
Ref. 80.9–154.3	3.2 g/day	123.7 ± 4.3	6 g/day	106.5 ± 3.2
	6 g/day	127.1 ± 4.9	12 g/day	113.0 ± 4.4
	9.2 g/day	128.5 ± 3.4	18 g/day	110.5 ± 2.6
	12 g/day	131.4 ± 5.3	24 g/day	106.2 ± 2.5
Lysine	Baseline	200.5 ± 5.0	Baseline	194.9 ± 4.9
Ref. 118.7–257.0	3.2 g/day	199.6 ± 5.1	6 g/day	171.1 ± 5.0*
	6 g/day	194.8 ± 6.6	12 g/day	165.8 ± 6.8**
	9.2 g/day	199.1 ± 4.6	18 g/day	153.6 ± 5.8**
	12 g/day	199.6 ± 6.0	24 g/day	146.4 ± 5.5**
Methionine	Baseline	27.4 ± 1.0	Baseline	26.8 ± 0.6
Ref. 19.2–32.7	3.2 g/day	27.0 ± 0.8	6 g/day	25.4 ± 0.9
	6 g/day	26.6 ± 1.2	12 g/day	25.3 ± 1.0
	9.2 g/day	27.7 ± 0.7	18 g/day	24.6 ± 0.8
	12 g/day	26.7 ± 0.9	24 g/day	22.7 ± 0.8**
Phenylalanine	Baseline	58.5 ± 1.6	Baseline	59.1 ± 2.2
Ref. 45.7–76.5	3.2 g/day	57.5 ± 1.5	6 g/day	58.0 ± 2.0
	6 g/day	58.0 ± 1.7	12 g/day	58.3 ± 2.4
	9.2 g/day	58.5 ± 1.3	18 g/day	58.3 ± 1.9
	12 g/day	56.6 ± 1.4	24 g/day	55.8 ± 1.6
Threonine	Baseline	134.9 ± 4.8	Baseline	138.7 ± 3.9
Ref. 89.2–205.0	3.2 g/day	128.8 ± 4.0	6 g/day	127.4 ± 5.6
	6 g/day	127.0 ± 4.0	12 g/day	126.8 ± 4.9
	9.2 g/day	128.9 ± 3.4	18 g/day	116.5 ± 4.1**
	12 g/day	126.9 ± 4.3	24 g/day	112.0 ± 4.0**
Tryptophan	Baseline	52.0 ± 1.6	Baseline	51.4 ± 1.6
Ref. 41.4–65.5	3.2 g/day	50.9 ± 1.4	6 g/day	49.5 ± 1.2
	6 g/day	50.5 ± 1.7	12 g/day	49.4 ± 1.5
	9.2 g/day	51.6 ± 2.0	18 g/day	49.6 ± 1.4
	12 g/day	50.1 ± 1.7	24 g/day	48.9 ± 1.6
Valine	Baseline	251.3 ± 13.3	Baseline	218.1 ± 5.7
Ref. 158.4–287.7	3.2 g/day	239.5 ± 8.1	6 g/day	204.7 ± 5.1
	6 g/day	246.4 ± 8.5	12 g/day	216.2 ± 8.1
	9.2 g/day	247.3 ± 5.9	18 g/day	206.7 ± 6.6
	12 g/day	257.0 ± 10.4	24 g/day	208.0 ± 5.8

NEAA, ornithine, and citrulline (μmol/L)	Ornithine (*n* = 23)		Citrulline (*n* = 23)	
Alanine	Baseline	367.9 ± 12.5	Baseline	366.6 ± 17.9
Ref. 239.9–510.2	3.2 g/day	376.6 ± 15.9	6 g/day	356.8 ± 18.8
	6 g/day	370.8 ± 20.6	12 g/day	353.6 ± 16.2
	9.2 g/day	385.6 ± 20.8	18 g/day	366.4 ± 15.2
	12 g/day	412.8 ± 22.9	24 g/day	371.7 ± 16.0
Arginine	Baseline	94.6 ± 3.6	Baseline	97.6 ± 2.9
Ref. 46.0–121.7	3.2 g/day	90.8 ± 3.5	6 g/day	114.4 ± 4.2
	6 g/day	90.7 ± 4.1	12 g/day	138.8 ± 7.9*
	9.2 g/day	91.4 ± 2.9	18 g/day	165.4 ± 11.3**
	12 g/day	93.8 ± 3.3	24 g/day	190.1 ± 14.4**
Aspartic acid	Baseline	2.4 ± 0.1	Baseline	2.5 ± 0.2
Ref. less than 3.2	3.2 g/day	2.6 ± 0.1	6 g/day	2.6 ± 0.1
	6 g/day	3.1 ± 0.1**	12 g/day	3.2 ± 0.177*
	9.2 g/day	2.9 ± 0.1*	18 g/day	2.8 ± 0.1
	12 g/day	2.5 ± 0.1	24 g/day	2.3 ± 0.1
Asparagine	Baseline	63.0 ± 1.4	Baseline	62.5 ± 1.1
Ref. 40.8–76.5	3.2 g/day	60.8 ± 1.3	6 g/day	57.3 ± 1.8
	6 g/day	61.1 ± 2.3	12 g/day	59.1 ± 1.6
	9.2 g/day	62.6 ± 1.8	18 g/day	58.3 ± 1.5
	12 g/day	64.6 ± 1.8	24 g/day	58.9 ± 1.4
Cystine	Baseline	44.9 ± 1.8	Baseline	43.5 ± 1.4
Ref. 36.5–56.0	3.2 g/day	46.0 ± 1.8	6 g/day	44.3 ± 1.3
	6 g/day	45.9 ± 1.5	12 g/day	44.3 ± 1.1
	9.2 g/day	46.1 ± 1.7	18 g/day	44.4 ± 1.2
	12 g/day	46.7 ± 1.7	24 g/day	45.8 ± 1.2
Glutamic acid	Baseline	28.0 ± 2.7	Baseline	26.0 ± 2.0
Ref. 10.8–44.4	3.2 g/day	41.6 ± 4.0	6 g/day	39.5 ± 2.3
	6 g/day	47.1 ± 4.6**	12 g/day	40.4 ± 3.3**
	9.2 g/day	39.5 ± 3.2	18 g/day	33.1 ± 2.1**
	12 g/day	35.2 ± 3.2	24 g/day	25.6 ± 2.1
Glutamine	Baseline	556.9 ± 11.0	Baseline	574.6 ± 12.2
Ref. 488.2–733.1	3.2 g/day	539.1 ± 11.0	6 g/day	524.0 ± 12.2*
	6 g/day	520.7 ± 10.9	12 g/day	509.7 ± 10.3**
	9.2 g/day	529.2 ± 12.1	18 g/day	514.7 ± 11.4**
	12 g/day	573.6 ± 11.1	24 g/day	554.1 ± 11.1
Glycine	Baseline	224.2 ± 6.9	Baseline	241.3 ± 8.7
Ref. 153.2–362.1	3.2 g/day	221.2 ± 6.5	6 g/day	226.0 ± 9.5
	6 g/day	216.1 ± 6.9	12 g/day	221.9 ± 8.7
	9.2 g/day	226.1 ± 6.7	18 g/day	224.4 ± 8.9
	12 g/day	221.9 ± 6.1	24 g/day	219.6 ± 8.0
Proline	Baseline	180.6 ± 20.8	Baseline	161.9 ± 8.5
Ref. 89.6–258.8	3.2 g/day	179.8 ± 20.2	6 g/day	156.6 ± 7.4
	6 g/day	187.0 ± 29.3	12 g/day	160.7 ± 11.1
	9.2 g/day	188.7 ± 29.2	18 g/day	149.8 ± 8.6
	12 g/day	194.1 ± 29.0	24 g/day	146.5 ± 8.3
Serine	Baseline	111.9 ± 3.4	Baseline	111.6 ± 3.4
Ref. 91.5–161.8	3.2 g/day	115.0 ± 3.3	6 g/day	109.0 ± 3.9
	6 g/day	115.7 ± 3.6	12 g/day	108.6 ± 4.3
	9.2 g/day	118.6 ± 3.4	18 g/day	106.3 ± 4.3
	12 g/day	115.3 ± 3.4	24 g/day	99.3 ± 3.5
Tyrosine	Baseline	59.9 ± 1.9	Baseline	59.3 ± 1.9
Ref. 50.2–82.6	3.2 g/day	57.8 ± 1.8	6 g/day	56.5 ± 2.0
	6 g/day	56.2 ± 2.2	12 g/day	54.9 ± 2.3
	9.2 g/day	59.7 ± 2.0	18 g/day	54.7 ± 1.6
	12 g/day	63.8 ± 2.3	24 g/day	55.9 ± 2.0
Ornithine	Baseline	49.4 ± 2.2	Baseline	49.2 ± 2.0
Ref. 43.2–95.7	3.2 g/day	54.7 ± 2.4	6 g/day	58.1 ± 2.6
	6 g/day	61.7 ± 6.5	12 g/day	66.1 ± 3.2*
	9.2 g/day	72.8 ± 6.1	18 g/day	73.5 ± 4.4**
	12 g/day	87.2 ± 10.4**	24 g/day	74.2 ± 5.5**
Citrulline	Baseline	29.9 ± 1.0	Baseline	31.5 ± 1.4
Ref. 20.4–44.8	3.2 g/day	29.0 ± 1.3	6 g/day	34.5 ± 1.4
	6 g/day	30.9 ± 1.3	12 g/day	40.0 ± 2.0
	9.2 g/day	29.4 ± 1.4	18 g/day	49.6 ± 3.6**
	12 g/day	29.6 ± 1.2	24 g/day	56.7 ± 4.7**

Values represent the mean and standers error (SE) (n = 23). One-way ANOVA was followed with the Tukey’s multiple comparisons test between each amino acid intake: *p < 0.05, **p < 0.01 from baseline

*EAA* essential amino acids, *NEAA* non-essential amino acids, *Ref.* reference range

### Adverse events

The number of adverse events observed from the onset of the event until the subject recovered from the event was defined as a single event. During the ORN supplementation, 16 adverse events were identified in 10 subjects. These included 5 events of gastrointestinal symptoms, 3 events of COVID-19 vaccination side effects, 2 event of common cold-like symptoms, and 1 event each of headache, fatigue, bone fracture, hematuria, exanthema, and drowsiness. Most of the events occurred during the initially tested low doses of ORN (3.2, 6, and 9.2 g/day). One event of gastrointestinal symptoms occurred at the highest dose (12 g/kg). Of all recorded events, 13 events were mild and required no treatment, and 3 were moderate and therefore required brief pharmacological treatment. One subject (ID13329) experienced a fracture, considered a serious condition as hospitalization was required. The occurrence of fracture was judged unrelated to ORN administration by investigator. Nevertheless, the subject requested to drop out of the study because of difficulties in complying with the supplementation protocol during hospitalization. One subject (ID13327) experienced hematuria (the first urine in the morning was darker than normal), and the study was discontinued at the discretion of the investigator, since the cause could not be identified from the laboratory data or by interviews with a doctor. This event was found to be unrelated to ORN supplementation, since it occurred only once, and the subject recovered quickly.

During the CIT supplementation study, 24 adverse events were identified in 11subjects. These included, 6 events of COVID-19 vaccination side effects, 4 events of headache, 3 events each of gastrointestinal symptoms, fatigue, arthralgia, and common cold-like symptoms, and 1 event each of giddiness and exanthema. Most of the events occurred during the initially tested doses (6 and 12 g/day). At the high doses (18 and 24 g/kg), 5 events of COVID-19 vaccination side effects and 1 event each of exanthema, gastrointestinal symptoms, and fatigue occurred. Of all recorded events, 17 events were mild and required no treatment, and 7 were moderate and therefore required brief pharmacological treatment. One subject (ID13378) reported giddiness, determined to be a serious potential adverse event. Low cerebrospinal fluid pressure was not diagnosed from the laboratory data or the interviews with a doctor. In addition, a causal relationship with the CIT supplementation was ruled out by the lead investigator. However, the subject requested to be dropped from the study. In all other reported events, neither ORN nor CIT supplementation was found to be causally related to non-continuous occurred events. Furthermore, all subjects have recovered after the study offset.

### Questionnaire regarding sleep quality and mental fatigue, and dietary intake estimation

Daily online surveys of subjective sleep quality and mental fatigue were conducted from 7 days prior to the start of intake of the tested amino acids until the end of the test period. The fatigue and sleep quality were survey using 11 graded score as changes from the previous day (best condition + 5, no change 0, and worst condition − 5). In CIT supplementation group, a statistically significant increase compared with baseline mental fatigue score (decrease fatigue) was observed at low test-dose (6 g/day). The score recovered during wash-out and no increase was observed at higher test-doses (Table 4). No significant effects of ORN at any tested dose, or CIT at 12, 18 and 24 g/day, on mental fatigue or sleep quality were found.

**Table 4:** Self-evaluated changes in sleep quality and mental fatigue during the oral ornithine or citrulline supplementation in this study

Ornithine (*n* = 23)	Sleep quality	Mental fatigue
Baseline (7 days)	0.019 ± 0.037	0.050 ± 0.051
3.2 g/day (28 days)	0.030 ± 0.049	0.095 ± 0.048
WO (14 days)	− 0.005 ± 0.049	− 0.018 ± 0.055
6 g/day (28 days)	0.043 ± 0.044	0.023 ± 0.051
WO (14 days)	0.000 ± 0.062	− 0.025 ± 0.070
9.2 g/day (28 days)	0.053 ± 0.046	0.031 ± 0.049
WO (14 days)	0.025 ± 0.047	0.022 ± 0.050
12 g/day (28 days)	0.023 ± 0.046	0.034 ± 0.045

Citrulline (*n* = 23)	Sleep quality	Mental fatigue
Baseline (7 days)	− 0.119 ± 0.089	− 0.108 ± 0.043
6 g/day (28 days)	0.066 ± 0.042	0.120 ± 0.045**
WO (14 days)	− 0.022 ± 0.024	− 0.006 ± 0.025
12 g/day (28 days)	0.087 ± 0.034	0.079 ± 0.031
WO (14 days)	0.019 ± 0.050	0.047 ± 0.034
18 g/day (28 days)	− 0.003 ± 0.040	0.038 ± 0.035
WO (14 days)	0.000 ± 0.046	0.012 ± 0.043
24 g/day (28 days)	0.030 ± 0.036	0.042 ± 0.023

Values represent the mean and standers error (SE) of all tested subjects (*n* = 23) over the whole period of supplementation (28 days), baseline (7 days), and wash-out recovery periods (14 days). One-way ANOVA was followed with Tukey’s multiple comparisons test between each amino acid intake, baseline, and wash-out period

**p < 0.01 from baseline

*WO* wash-out period

A dietary intake survey was conducted at the end of each 4-week period using the brief-type self-administered diet history questionnaire (BDHQ) food habit assessment (Miura et al. 2021), which was previously validated for Japanese subjects (Sasaki et al. 2003). No significant effects of either ORN or CIT supplementation on intake of total energy (kcal/day), protein (g/day), fat (g/day), or carbohydrates (g/day) were found (Table5). Similarly, no ORN or CIT administration-related changes in subject characteristics (BW, BMI, SAP, DBP, and HR) were found when compared to the pre-intervention data (Table 5).

**Table 5:** Characteristics and BDHQ data on end of each term

Ornithine (*n* = 23)	Baseline	3.2 g/day	6 g/day	9.2 g/day	12 g/day
BW (Kg)	68.6 ± 1.7	68.5 ± 1.8	68.1 ± 1.8	67.7 ± 1.8	68.2 ± 1.8
BMI (kg/m2)	23.8 ± 0.5	23.7 ± 0.6	23.6 ± 0.6	23.4 ± 0.6	23.6 ± 0.6
SAP (mmHg)	121.7 ± 2.3	121.8 ± 2.1	119.1 ± 2.3	117.3 ± 2.1	122.8 ± 2.1
DBP (mmHg)	73.7 ± 2.1	70.0 ± 2.1	68.3 ± 2.0	67.0 ± 1.5	72.7 ± 2.0
HR (mmHg)	67.5 ± 2.2	67.4 ± 2.4	68.3 ± 2.4	66.9 ± 2.5	65.7 ± 2.0
Total energy (kcal/day)	1762 ± 132	1643 ± 105	1906 ± 151	1697 ± 116	1597 ± 111
Protein (g/day)	61.5 ± 5.0	59.0 ± 3.6	62.8 ± 5.3	61.4 ± 4.7	56.0 ± 3.9
Fat (g/day)	49.5 ± 3.7	49.9 ± 3.1	52.7 ± 4.5	50.7 ± 3.6	48.5 ± 2.6
Carbohydrates (g/day)	236.2 ± 20.5	213.8 ± 17.1	260.7 ± 22.7	218.4 ± 17.9	204.1 ± 17.7

Citrulline (n = 23)	Baseline	6 g/day	12 g/day	18 g/day	24 g/day
BW (Kg)	70.5 ± 1.7	70.0 ± 1.7	69.5 ± 1.7	69.4 ± 1.6	70.3 ± 1.6
BMI (kg/m2)	23.5 ± 0.5	23.3 ± 0.5	23.2 ± 0.5	23.1 ± 0.5	23.4 ± 0.5
SAP (mmHg)	122.0 ± 2.0	121.3 ± 2.4	116.8 ± 2.1	120.1 ± 2.9	123.1 ± 2.9
DBP (mmHg)	74.2 ± 1.7	69.7 ± 2.1	67.4 ± 1.9	69.4 ± 1.8	71.1 ± 2.1
HR (mmHg)	67.7 ± 1.8	69.0 ± 1.7	71.0 ± 2.0	67.4 ± 1.8	67.0 ± 1.8
Total energy (kcal/day)	1493 ± 115	1602 ± 101	1657 ± 115	1692 ± 118	1731 ± 114
Protein (g/day)	57.2 ± 4.8	59.9 ± 4.0	63.0 ± 7.1	65.5 ± 5.8	68.0 ± 5.7
Fat (g/day)	47.6 ± 4.6	49.0 ± 3.7	48.5 ± 3.9	52.4 ± 4.4	56.6 ± 4.3
Carbohydrates (g/day)	195.7 ± 17.6	208.8 ± 15.8	223.6 ± 16.6	219.4 ± 17.0	218.2 ± 16.9

Values represent the mean and standers error (SE) (*n* = 23). One-way ANOVA was followed with the Tukey’s multiple comparisons test between each amino acid intake

*BW* body weight, *BMI* body mass index, *SAP* systolic arterial pressure, *DBP* diastolic blood pressure, *HR* heart rate

## Discussion

To the best of our knowledge, these are the first dedicated dose–response subchronic clinical safety studies reported for either ORN or CIT. The results indicated that the subchronic clinical NOAEL for ORN and CIT is at the highest dose tested (12 g/day for ORN and 24 g/day for CIT). The NOAEL for ORN is comparable to the previously described acute upper limit of intake for CIT (15 g/day, Moinard et al. 2008). There is no published dose-dependent clinical safety study with CIT, and therefore, a direct comparison is not possible. Rodent subchronic toxicology tests of both ORN and CIT have been published (Ishida et al. 2013; Morita et al. 2017), but for nutrients ingested daily from regular foods at doses equal or higher than 1 g, such as amino acids, rodent toxicology test results have limited implications to human risk assessment (e.g., Miura et al. 2021). Therefore, the interpretation of the current trials includes human data only.

ORN did not trigger a change in blood parameters at any of the test-doses. In contrast, supplemental CIT (6 and 9.2 g/day) slightly increased serum levels of lactate dehydrogenase, but these changes had neither a dose-dependent character nor a pathological significance within the reference range of lactate dehydrogenase and thus could have been non-specific outcomes due to chance alone.

ORN and CIT are involved in the urea cycle (ORN cycle), which plays an important role in detoxification of ammonia (Wu 2021). In the urea cycle, ORN is synthesized from arginine by arginase, and metabolized to CIT by ORN transcarbamylase. ORN supplementations were associated with changes in plasma levels of aspartic acid, glutamic acid, glutamine, and ORN, although the changes were only marginal for aspartic acid and glutamic acid for mid-doses ORN supplementations. CIT supplementation was associated with changes in plasma levels of arginine, aspartic acid, glutamic acid, glutamine, lysine, methionine, threonine, ORN, and CIT, whereas ORN supplementation was not associated with changes in the plasma levels of any amino acid at any tested dose.

**Fig. 2:**
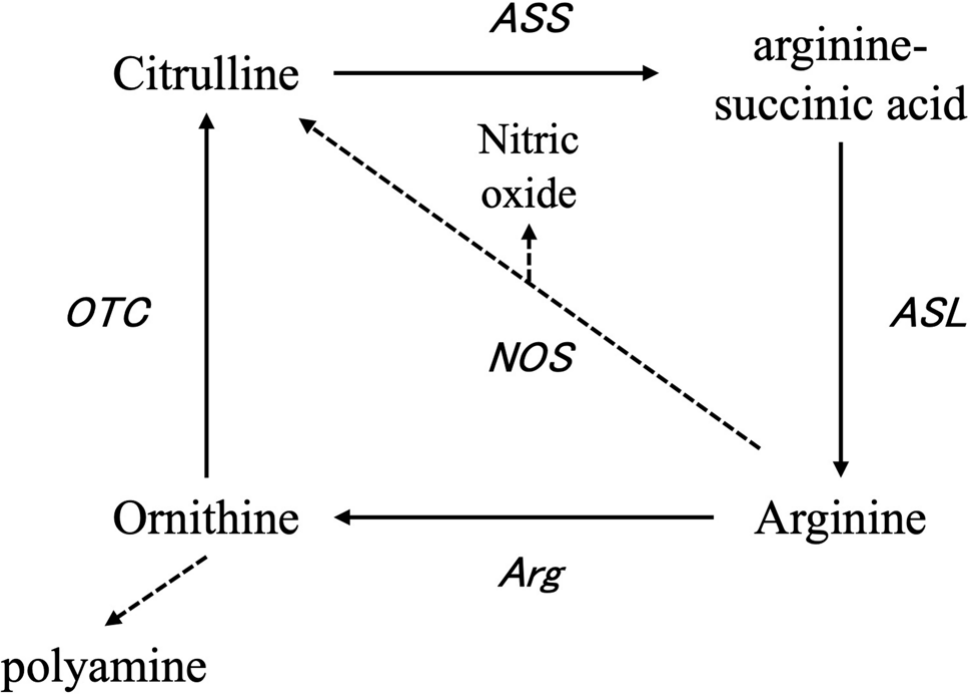
Schematic representation of the ornithine–citrulline—arginine-succinic acid—arginine metabolic pathway (ornithine cycle). The abbreviations in the table are indicated by ornithine transcarbamylase (OTC), arginosuccinate synthase (ASS), arginosuccinate lyase (ASL), arginase (Arg), and nitric oxide synthase (NOS)

CIT is a well-known precursor for arginine production (Hecker et al. 1990; Wu and Brosnan 1992; Romero et al. 2006) and plasma arginine increases following oral CIT supplementation (Suzuki et al. 2017), which agrees with the results of the present study. CIT administration triggered small but statistically significant decrease in the plasma concentrations of lysine (CIT doses, 6, 12, 18, and 24 g/day), methionine (CIT doses, 24 g/day) and threonine (CIT doses 18 and 24 g/day) plasma levels when compared to the baseline values. These changes were within the reference ranges of the clinic and were not found to be pathologically relevant. Mid-dosages CIT supplementation was associated with marginal changes in plasma levels of aspartic acid (CIT doses, 12 g/day), glutamic acid (CIT doses, 12 and 18 g/day), and glutamine (CIT doses, 6, 12 and 18 g/day). The change in plasma CIT levels was a direct consequence of the supplementation, and the change in plasma ORN levels was considered to be an outcome of the metabolism from CIT via arginine-succinic acid and from arginine by arginine-succinate synthase, arginine-succinate lyase, and arginase 1 in the ornithine cycle (Fig. 2).

There were no observed changes in the questionnaire scores of sleep quality and mental fatigue at any tested doses without CIT (6 g/day). The observation was decrease fatigue (increase of mental fatigue score) at CIT (6 g/day). This result was consistent with Bendahan et al.’s reported study results of decrease fatigue. Since supplementation of oral CIT may have improved fatigue levels through enhanced exercise performance (Bendahan et al. 2002). On the other hand, the decrease fatigue at the CIT (6 g/day) was lack of dose-dependency and recovery of wash-out term. This event requires further detailed study. Therefore, the decrease fatigue at the 6 g/day of CIT was not deemed treatment-related from investigator on the safety study. A range of mild adverse effects, mainly gastrointestinal or side effects of COVID-19 vaccination, were described by subjects in both the ORN and CIT groups. One of the possible causes of gastrointestinal symptoms may be the non-specific and non-continuously gastrointestinal effects of taking cellulose capsules. Because the tests were conducted without placebo controls, it was impossible to discriminate the non-specific gastrointestinal effects of ingesting cellulose capsules from the effects of the tested amino acids. Non-gastrointestinal adverse effects (headache, tiredness, and cold-like symptoms) were mild, infrequent, and happened mostly in the initial phases of the trial. Therefore, they were not considered to be causally linked to the supplemented amino acids. ORN, CIT, and arginine are closely related in metabolism via the ornithine cycle (Fig. 2). Since it is important to compare the safe intake dose (NOAEL) of these three amino acids. In this study (4 weeks) and Mc Neal et al. reported (90 days), CIT (12, 18, 24 g/day intake) increases plasma arginine and ORN concentrations, CIT (18, 24 g/day intake) increases plasma CIT concentrations, CIT (12 g/day intake) increases ORN blood concentrations, and arginine (15, 30 g/day intake) increases plasma arginine and ORN concentrations. And safe intakes’ dose (NOAEL) is ORN (12 g/day intake), CIT (24 g/day intake), and arginine (30 g/day intake), respectively. Since the three amino acids have different functionalities as food components, there was a possibility that there would be differences in safety, but this study has revealed that there are no health problems even if relatively large amounts of any of the amino acids are ingested.

The duration of supplementation (4 weeks) was chosen based on previous clinical studies (Deutz et al. 2017; Gheller et al. 2020; Miura et al. 2021; McNeal et al. 2018) as both the most appropriate and practical period to evaluate tolerance to dietary intake of amino acids. In the study protocol, blood biochemical tests were taken under fasting conditions for a minimum of 8 h. Therefore, this methodology did not allow the quantification of the acute effects on blood biochemistry. There were three other limitations to the present study. First, no attempt was made to test-doses higher than 12 g/day of ORN or 24 g/day of CIT, and no clear marker of toxicity was found. The decision not to increase the dose further was based on ethical concerns with requesting the subjects to ingest more than 60 capsules daily for a prolonged time, and this study already used doses above the practical dose for supplementation (Moinard et al. 2008; Sugino et al. 2008; Dietary Supplement Label Database 2022). Second, it is not possible to discern if the presence of hydrochloride in the ORN supplementation compound (ORN hydrochloride) had any effect on the observed circulating parameters. However, the hydrochloride form is typically used in supplements, and therefore, it was chosen for the study. Third, the study was limited to male subjects of a single ethnic group.

## Conclusion

The present results obtained in healthy male adults allow the authors to propose the highest tested dose (12 g/day and 24 g/day) as a subchronic clinical NOAEL for supplemental ORN hydrochloride and CIT, respectively.

## Data Availability

Available on request.
